# Role of Semaphorins in Immunopathologies and Rheumatic Diseases

**DOI:** 10.3390/ijms20020374

**Published:** 2019-01-16

**Authors:** Samuel Garcia

**Affiliations:** Department of Rheumatology and Clinical Immunology and Laboratory of Translational Immunology, University Medical Center Utrecht, University of Utrecht, Heidelberglaan 100, 3584 CX Utrecht, The Netherlands; s.garciaperez@umcutrecht.nl; Tel.: +31-(0)88-75-54597

**Keywords:** rheumatic diseases, semaphorins, therapeutic targets

## Abstract

Rheumatic diseases are disorders characterized by joint inflammation, in which other organs are also affected. There are more than two hundred rheumatic diseases, the most studied so far are rheumatoid arthritis, osteoarthritis, spondyloarthritis, systemic lupus erythematosus, and systemic sclerosis. The semaphorin family is a large group of proteins initially described as axon guidance molecules involved in nervous system development. Studies have demonstrated that semaphorins play a role in other processes such as the regulation of immunity, angiogenesis, bone remodeling, apoptosis, and cell migration and invasion. Moreover, semaphorins have been related to the pathogenesis of multiple sclerosis, asthma, Alzheimer, myocarditis, atherosclerosis, fibrotic diseases, osteopetrosis, and cancer. The aim of this review is to summarize current knowledge regarding the role of semaphorins in rheumatic diseases, and discuss their potential applications as therapeutic targets to treat these disorders.

## 1. Introduction

Rheumatic diseases are characterized by inflammation that affects the connecting or supporting structures of the body. The most commonly affected are the joints, other structures include tendons, ligaments, bones, and muscles. These rheumatic diseases also affect other organs such as skin, eyes, intestinal tract, lungs, kidney, heart, and brain. There are more than two hundred rheumatic diseases, in this review we focus on rheumatoid arthritis (RA), osteoarthritis (OA), spondyloarthritis (SpA), systemic lupus erythematosus (SLE), and systemic sclerosis (SSc) [1,2]. These disorders incapacitate patients, leading to loss of quality of life and significant socio-economic costs. In fact, rheumatic diseases account for 21.3% of total years lived with disability, the second after mental and behavioral problems [3].

The semaphorin family is a large group of proteins initially described as regulators of nervous system development. Members of the semaphorin family are classified into eight categories: classes 1 and 2 are found in invertebrates, classes 3–7 are found in vertebrates, and class 8 are viral-encoded proteins. Among the vertebrate semaphorins, class 3 members are secreted, and those in classes 4–7 are membrane-attached. However, some of the membrane-bound semaphorins can be cleaved and released into the circulation. A structural hallmark of all semaphorins is the N-terminal ~500-residue-long sema domain, consisting of a seven-blade β-propeller fold. Semaphorins are also defined by their PSI (plexins, semaphorins, and integrins), immunoglobulin-like (Ig), and basic C-terminal domains [4,5,6,7]. Main receptors for semaphorins are the plexins, a family comprising four classes (PlexinA–D) and nine members in vertebrates. The extracellular domain of plexins also includes a sema domain that is putatively involved in ligand binding. In addition to the PlexinA members (Plexin A1–A4), which act as signal transducing motifs, class 3 semaphorins also require the co-receptors neuropilins (NRP-1 and -2), which constitute the ligand-binding subunit. In addition, some semaphorins can bind to other receptors, such as TIM-2 (Sema4A), CD72 (Sema4D), β1-integrin (Sema7A), and MET (Sema4D and Sema5C) [4,5,8,9,10].

Besides their role in controlling cell migration and axonal growth cone guidance, studies have shown that semaphorins play a role in other biological pathological processes, including inflammation, angiogenesis, apoptosis, fibrosis, bone remodeling, and cell invasion [6,7,11,12,13,14,15]. Many of these processes are involved in cancer pathology, suggesting semaphorins could be promising therapeutic targets in different types of tumors. However, the importance of semaphorins in cancer has been extensively studied, and it is not the aim of this review [7,12,14,16,17,18,19,20]. Semaphorins have also been implicated in the pathology of other diseases, including multiple sclerosis, asthma, Alzheimer, myocarditis, atherosclerosis, fibrotic diseases, osteopetrosis, and multiple types of cancer [4,5,6,7,13,14,15,16,19,21,22,23].

The aim of this review is to summarize the role of semaphorins in rheumatic diseases and discuss their potential use as therapeutic targets.

## 2. Rheumatoid Arthritis

Rheumatoid arthritis is a complex autoimmune disease that mainly affects peripheral joints, causing the destruction of joint structure, and ultimately joint deformity and disability. RA can also affect other organs including skin, lung, heart, and vasculature, which contribute to the morbidity and mortality. In fact, several studies have shown that life expectancy is reduced by 3–10 years in RA patients [1,2,24,25]. RA is characterized by the activation and infiltration of immune cells in the affected joints, mainly monocytes/macrophages, B cells, and T cells. This is also mediated by the activation and proliferation of the fibroblast-like synoviocytes (FLS), which are the joint stromal cells. Activated immune cells and FLS release inflammatory cytokines, chemokines, growth factors, adhesion molecules, and degrading enzymes (matrix metalloproteinase and agrecanasses), which perpetuate chronic inflammation, promote the hyperplasia of the synovial membranes, induce the formation of new blood vessels from the existing vasculature, and the destruction of joint structures [24,25,26,27,28,29,30].

Recently, the implication of semaphorins in the pathology of RA has been extensively studied, with class 3 being the most studied semaphorin family. Initial studies showed that Sema3C expression is elevated in the synovial tissue of RA patients compared to OA patients and control individuals, suggesting an implication in disease pathology [31]. However, the functional role of Sema3C in RA is unknown. Several studies have demonstrated that synovial tissue and serum expression of Sema3A is reduced in RA patients compared to healthy controls (HC) and OA patients. Moreover, Sema3A expression is negatively correlated with disease activity parameters, such as Disease Activity Score 28 (DAS28), C-reactive protein (CRP), and presence of the Rheumatoid Factor (RF). These studies have shown that the induction of Sema3A expression reduced the severity of arthritis in collagen-induced arthritis (CIA), and K/BxN serum-transfer induced arthritis mouse models. In vitro experiments suggest that this protective effect might be due to different mechanisms, such as the reduction of inflammatory mediators production by CD4^+^ T cells (interferon-γ (IFN-γ) and interleukin-17 (IL-17)) and bone marrow derived macrophages (inducible Nitric Oxide Synthases (iNOS), tumor necrosis factor α (TNFα), IL-1β, and IL-6), the induction of the anti-inflammatory cytokine IL-10 by CD4^+^ T cells, and the inhibition of osteoclastogenesis. Sema3A also showed an anti-inflammatory effect in primary human cells and its administration reduced the expression of inflammatory cytokines by peripheral blood mononuclear cells (PBMCs) (TNFα, IL-23, IL-6) and CD4^+^ T cells (IFN-γ, TNFα and IL-6), and induced the expression of IL-10 by CD4^+^NRP1^+^ T cells. Sema3A also inhibited the proliferative and migratory ability of endothelial cells, suggesting an anti-angiogenic role [32,33,34]. Finally, Sema3A is involved in the viability, in addition to the migratory and invasive ability of RA FLS, although results are contradictory. Results from our group showed that Sema3A does not affect cell viability and proliferation, but induces RA FLS migration and invasion. However, other studies have shown that Sema3A induces RA FLS apoptosis, and reduces vascular endothelial growth factor_165_ (VEGF_165_)-mediated cell migration and invasion [34,35].

Previously published results from our group have shown that synovial tissue and synovial fluid expression of Sema3B, Sema3F, and Sema3G is reduced in RA compared to undifferentiated arthritis (UA) patients. In addition, Sema3B, Sema3F, and Sema3G expression was negatively correlated with DAS28, the swollen joint count of 28 joints (SJC28), and the tender joint count of 28 joints (TJC28) scores; in addition to the clinical parameters CRP, erythrocyte sedimentation rate (ESR), and the synovial mRNA expression of IL-6, TNFα, and IL-1β. Importantly, IL-1β and TNFα, the two major cytokines involved in RA pathogenesis, reduce the expression of Sema3B and Sema3F by RA FLS, which is the cell type responsible for the production of these semaphorins in the synovium. These results suggest that deregulation of class 3 semaphorins might be implicated in the pathology of RA. In fact, we have also demonstrated that Sema3B and Sema3F do not affect the viability and proliferation of RA FLS, but reduce their migratory and invasive capacities. These effects were associated with the down-regulation of matrix metalloproteinases (MMP)-1 and -3 expression and were mediated by the PlexinA1 and neuropilin-1 and -2 receptors [35]. Therefore both Sema3B and Sema3F might protect against progressive joint destruction in RA.

The two most studied class 4 semaphorins, Sema4A and Sema4D, have been associated with pathology of RA. Sema4A is elevated in the synovium, synovial fluid, and serum of RA compared to OA patients. Importantly, synovial fluid and serum levels of Sema4A positively correlate with the DAS28 score and with the serum levels of the inflammatory mediators TNFα and IL-6, respectively [36]. Functional studies demonstrated that Sema4A plays a dual role in RA FLS. On one hand, Sema4A promotes inflammation through the induction of IL-6 expression by RA FLS. On the other hand, it enhances the invasive ability of RA FLS and induces the expression of the matrix metalloproteinases MMP-3 and MMP-9, all these effects are mediated by PlexinD1 [36]. Sema4D expression is higher in the synovium and synovial fluid of RA compared to OA patients, and serum levels are also elevated in RA patients compared to HC and OA, AS and SLE patients. Similarly to Sema4A, serum levels of Sema4D positively correlate with the DAS28 score [37]. Functional experiments showed that Sema4D induced the production of IL-6 and TNFα by CD14^+^ monocytes. More importantly the administration of an anti-Sema4D antibody reduced the severity of arthritis, and led to the production of inflammatory cytokines in the CIA mouse model [37].

A recent study has demonstrated that Sema5A expression is also higher in the serum of RA patients compared to HC, SLE, and Sjögren syndrome (SS) patients. Sema5A induces cell proliferation and production of pro-inflammatory cytokines by T and natural killer (NK) cells from HC, and enhances the effects induced by IL-2/IL-15 stimulation. Sema5A also induced the skewing of CD4^+^ T cells into Th1/Th17 subsets. As Th1/Th17 cells and elevated cytokine levels (TNFα, IL-1β, IL-6, and IL-8) induced by Sema5A play an essential role in the pathogenesis of RA, these data suggest that secreted Sema5A may contribute to the development and progression of the disease [38].

Finally, Sema7A levels are elevated in the synovial fluid and serum of RA patients compared to HC and OA patients, and these levels positively correlate with the DAS28 score, the RF, and CRP levels. In vitro data show that Sema7A induces the expression of TNFα and IL-6 by CD14^+^ monocytes in a β1-integrin-dependent manner. It also induces the expression of the transcription factors T-bet and RAR-related orphan receptor (ROR)γt by CD4^+^ T cells, suggesting that Sema7A might be implicated in the differentiation of Th1 and Th17 cells. In vivo data support this hypothesis, as the blocking of Sema7A attenuates severity of the arthritis and the serum levels of IL-17, TNFα, and IL-6 in the CIA mouse model [39].

**Figure 1:**
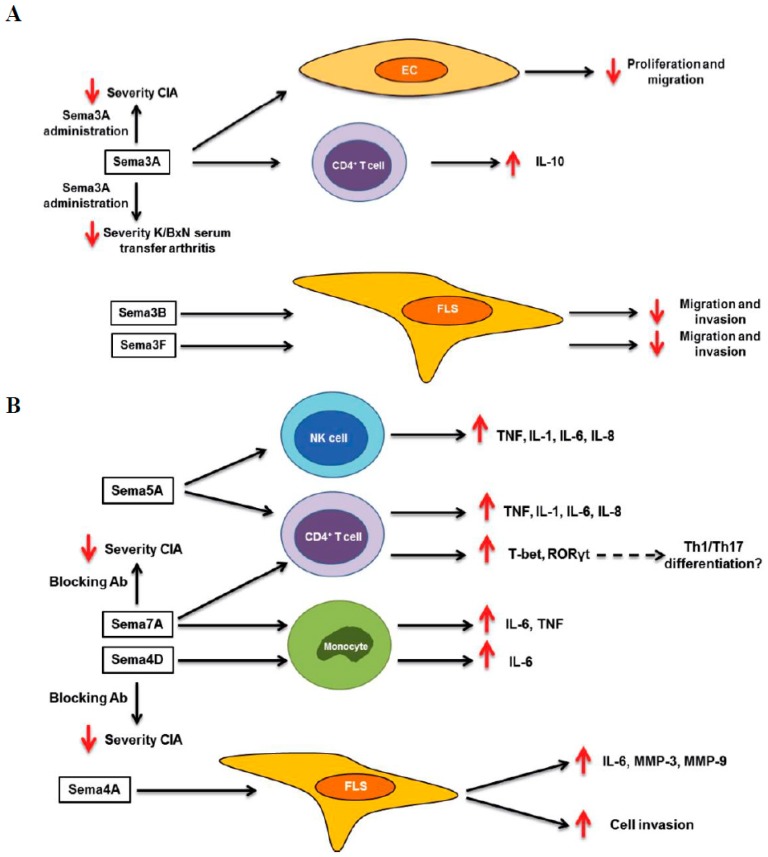
Role of semaphorins in rheumatoid arthritis. Schematic overview of the role of elevated (**A**) and reduced (**B**) semaphorins in the pathology of rheumatoid arthritis RA. Up red arrows and down red arrows indicate induction or reduction, respectively. CIA (collagen-induced arthritis). EC (endothelial cell).

Overall, these studies suggest that semaphorins may be useful biomarkers in RA, mainly for the measurement of disease activity. However, validations in larger cohorts are needed for their future use as biomarkers. More importantly, semaphorins play roles in different processes involved in the pathogenesis of RA, like immune activation, cytokine production, apoptosis, cell proliferation, cell migration and invasion (Figure 1). Therefore targeting these members, specific semaphorins, may be a therapeutic tool for disease treatment. In the case of Sema3B and Sema3F, they have a protective role, and therefore, their administration might impair joint destruction in RA patients. The rest of semaphorins are implicated in the development and progression of disease, and neutralizing their activity would be needed for a therapeutic use. However, further in vitro experiments and in animal models of arthritis are needed before pre-clinical testing and clinical trials.

## 3. Systemic Sclerosis

Systemic sclerosis is a severe autoimmune inflammatory disease of unknown etiology, with high morbidity and mortality rates. In fact, SSc is recognized as the most severe connective tissue disorder, associated with the highest case-specific mortality among all rheumatic diseases, and for which hardly any therapeutic options are available. SSc is characterized by activation of the immune system, vascular abnormalities, fibrosis triggering in skin thickening, and stiffness and loss of internal organ function that leads to profound disability and premature death [40,41,42,43,44].

Multiple studies have shown that immune cells play an important role in SSc pathology, where deregulation of the immune response is a hallmark of disease. Some of the consequences of this immune deregulation, include: the presence of auto-antibodies, mainly antinuclear (ANA), anticentromere (ACA), and antitopoisomerase (Scl70); the activation of circulating monocytes and the elevated infiltration of macrophages in the affected tissue of SSc patients; and altering of T cell homeostasis, leading to the elevated frequency of Th2 and Th17 cells observed in the peripheral blood and skin of SSc patients [40,41,45,46,47,48,49]. Vascular injury is an early event in scleroderma. It precedes fibrosis and involves small vessels, particularly the arterioles. The loss of arteries and capillaries is observed in many organs, including the skin. However, despite the loss of vasculature and that pro- and anti-angiogenic factors are overexpressed in SSc skin and serum, compensatory angiogenesis is deregulated and does not occur normally [40,41,43,50]. Fibrosis is a cardinal feature of SSc pathology characterized by excessive deposition of extracellular matrix (ECM) and proteins including collagen and fibronectin, as well as increased numbers of fibroblasts expressing the contractile protein α smooth muscle actin (α-SMA) within the interstitial spaces of tissues [40,41,43,51,52].

Similarly to RA and SLE, class 3 semaphorins are the most studied semaphorin family in SSc pathogenesis. Rimar D et al. showed that Sema3A expression is reduced in serum and regulatory T cells (Treg) from SSc patients compared to HC [53]; however, Romano E et al. did not replicate this finding, as they did not find differences in the serum and skin expression of Sema3A between HC and SSc patients [54]. These differences may be due to disease heterogeneity or to the small number of patients analyzed. Therefore, the role of Sema3A in SSc remains unclear and further investigations are needed. On the contrary to Sema3A, serum levels and skin expression of Sema3E are elevated in SSc patients compared to HC. The consequences of this elevated expression may be related to the vascular abnormalities observed in SSc, as Sema3E reduces in vitro angiogenesis, and this effect was abrogated after the blocking of PlexinD1, the receptor of Sema3E. Interestingly, serum of SSc patients also reduced angiogenesis, and this effect was abolished after blocking PlexinD1, supporting the anti-angiogenic role of Sema3E [55].

**Figure 2:**
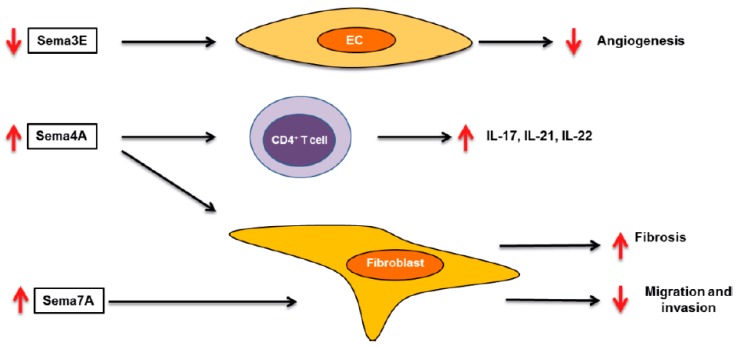
Role of semaphorins in systemic sclerosis. Schematic overview of the role semaphorins in the pathology of systemic sclerosis SSc. Up red arrows and down red arrows indicate induction or reduction, respectively. EC (endothelial cell).

Class 4 semaphorins have been also studied in the context of SSc. A descriptive work showed that Sema4D expression is higher in CD4^+^ T cells of SSc patients compared to HC, and this expression was associated with clinical characteristics such as anti-antibody levels, skin thickening, and inflammatory status [56]. Sema4A might also play a role in SSc pathology, as Sema4A induces collagen contraction by SSc patient lung fibroblasts [57]. Moreover, we have found that plasma levels of Sema4A, as well Sema4A expression by circulating monocytes and CD4^+^ T cells, were significantly higher in SSc patients compared to healthy controls. Functional assays showed that Sema4A enhanced the expression of Th17 cytokines induced by CD3/CD28 in CD4^+^ T cells, effects of which were abrogated by blocking or silencing Sema4A receptors PlexinD1, PlexinB2, and NRP-1. Sema4A also induced a pro-fibrotic phenotype in dermal fibroblasts from both HC and SSc patients, and this effect was mediated by PlexinD1 and PlexinB2. Therefore, we demonstrated that Sema4A plays an important and dual role in SSc pathology, through the promotion inflammation and fibrosis, the two main features of SSc, suggesting that Sema4A might be a novel therapeutic target in SSc (manuscript under review). Sema7A is also elevated in SSc patients, in the subgroup of patients with interstitial lung involvement [58]. Several works have shown that mice deficient in Sema7A have lower disease severity in different models of fibrosis, thus suggesting that Sema7A plays a pro-fibrotic role in the pathology of SSc [59,60]. Therefore, these semaphorins play important roles in the pathogenesis of SSc (Figure 2) and might be interesting therapeutic targets.

## 4. Osteoarthritis

Osteoarthritis is a late-onset disease characterized by the loss of articular cartilage of the synovial joints, principally from the hands, hips, and knees. OA is the most common rheumatic disease, as ~80% of people older than 75 years present progressive cartilage degradation. Although the loss of articular cartilage is a prominent feature during the evolution of OA, it is now commonly accepted that other joint tissues, such as subchondral bone and the synovial membranes, are also affected. The pathologic changes seen in OA joints include degradation of the articular cartilage, thickening of the subchondral bone, formation of osteophytes, inflammation of the synovium, and hypertrophy of the joint capsule [61,62,63,64]. Chondrocytes are the most important cell type involved in OA pathology and they undergo phenotypic changes during disease progression. These changes include cell proliferation, cluster formation, and increased production of both matrix proteins and matrix-degrading enzymes. Other cell types, including infiltrating immune cells and FLS have been associated with the pathology of OA. These cells produce cytokines such as TNFα, IL-1β, IL-15, and IL-17, which can perpetuate the inflammatory process, suppress matrix synthesis, and promote cartilage catabolism [62,65].

Despite the prominent roles of semaphorins in pathological processes implicated in OA, the potential alterations and functions of this family in the disease are largely unknown. Recent works have shown that Sema3A is elevated in the cartilage of OA patients compared to healthy controls, and that TNFα and IL-1 induce Sema3A production by a mouse chondrocyte cell line (ATDC5) and rat primary chondrocytes. In ATDC5 cells, Sema3A induced apoptosis in a phosphatidylinositide 3-kinase/protein kinase B (PI3K/PKB)-dependent manner. In human chondrocytes from OA patients, Sema3A inhibited the VEGF_165_-induced chondrocyte cloning, a process involved in the repair of the damaged cartilage [66,67]. These results suggest that Sema3A inhibition could be an interesting therapeutic target for the treatment of OA. However, Sema3A can also have protective roles in the disease. In fact, Sema3A can reduce the expression of IL-1β, TNFα, cyclooxygenase (COX)-2, MMP-3, and MMP-13 induced by mechanical stress in ATDC5 cells [68]. Moreover, Hayashi M et al. demonstrated that Sema3A is a potent osteoprotective factor by inhibiting bone resorption and promoting bone formation [69]. Therefore, further studies in vitro and in vivo are needed for elucidating the role of Sema3A in OA pathogenesis.

## 5. Systemic Lupus Erythematosus

Systemic lupus erythematosus is a heterogeneous and complex autoimmune inflammatory disease that predominately affects women of reproductive age. Although skin, kidneys, and joints are the main targeted organs, SLE can affect almost any organ. The pathogenesis of SLE is characterized by the formation of autoantibodies, the formation and deposition of immune complexes, and a breakdown of the immune tolerance of the body, which lead to systemic inflammation and tissue injury [70].

Sema3A is also implicated in the pathogenesis of SLE, and its expression is reduced in the serum of SLE patients compared to RA patients and healthy controls. In addition, Sema3A levels negatively correlate with the Systemic Lupus Erythematosus Disease Activity Index (SLADAI), and lower serum levels of Sema3A are associated with kidney involvement and presence of anti-cardiolipin antibodies, which are risk markers for thrombotic events in SLE patients [71]. In contrast, the expression of Sema3A was higher in the renal biopsies taken from lupus nephritis patients compared to control individuals [72]. However, this discrepancy may be due to the impaired ability of the kidneys to secrete semaphorins during advanced renal damage, because Sema3A expression negatively correlates with the renal function. The expression of Sema3A is also reduced in CD19^+^ CD25^high^ regulatory B (Breg) cells of SLE patients. Interestingly, Sema3A stimulation induces the expression of FoxP3 in Breg cells from HC and SLE patients, but reduces the expression of Toll-like receptor 9 (TLR9) by total B cells of SLE patients, suggesting an immunoregulatory role of Sema3A in the pathology of the disease [71,73]. This protective role was confirmed in vivo, as Sema3A administration decreased the glomerular inflammation and immune complex deposition, and prolonged survival in a mouse model of SLE [74]. Altogether, these results suggest that Sema3A may be an interesting therapeutic approach in SLE pathology.

In contrast to Sema3A, Sema5A is elevated in the serum and PBMCs of SLE patients compared to HC, and serum levels were positively correlated with SLEDAI score and other clinical parameters as proteinuria excretion and CRP. Moreover, patients with higher levels of Sema5A show a higher incidence of rash, serositis, and nephritis [38]. Therefore, this work suggests that Sema5A might be implicated in the pathology of the disease, although the functional role has not been elucidated yet.

Finally, the expression of Sema4A was higher in the glomeruli and tubuli of lupus nephritis patients compared to HC, although the functional consequences are unknown [72].

## 6. Spondyloarthritis

Spondyloarthritis is a polygenic rheumatic disease characterized by inflammation of the spine and peripheral joints, as well as extra-articular manifestations, such as inflammation in the intestinal tract, eyes, and skin. Spondyloarthritis comprises differently related but phenotypically distinct disorders, such as psoriatic arthritisarthritis related to inflammatory bowel disease; reactive arthritis, a subgroup of juvenile idiopathic arthritis; and ankylosing spondylitis, which is the prototypic and best-studied subtype [2,75,76,77]. RA and SpA are clinically distinct diseases with different etiologies, both are characterized by synovial hyperplasia, elevated synovial expression of many proinflammatory cytokines including TNFα, IL-1β, and IL-6, increased angiogenesis, and eventual joint destruction [24,76,78,79]. However, the potential role of semaphorins in the pathogenesis of SpA is completely unknown. In fact, only a couple of studies have investigated the expression of Sema3A in SpA patients, which show different results. Perrota et al. did not find differences in serum levels of Sema3A between HC and SpA patients [80]; however Liao HT et al. found higher levels in SpA patients [81]. Therefore more studies are needed for elucidating the potential role of Sema3A and other semaphorins in SpA pathology.

## 7. Concluding Remarks and Future Perspectives

In this review we have summarized the role of semaphorins in rheumatic diseases. Although in the last years the number of studies concerning the role of semaphorins in these diseases has exponentially increased, so far only a few members of the family have been implicated in disease pathology and are potential therapeutic targets. The discovery of new therapies is essential in these rheumatic diseases, because effective therapies are lacking or are only effective in some patients. In fact, even in RA, which is the best characterized disease, and most of the patients respond to the current therapies where reduced disease progression is achieved, the response is variable and nearly one-third of RA patients fail to enter clinical remission [82]. The need of therapeutic options is more pronounced in other disease such as SSc, for which a true therapy is lacking, and available therapies can only treat organ manifestations [42].

The therapeutic approach would be different depending on the semaphorin. Sema3A, Sema3B, and Sema3F administration could be a therapeutic option for the treatment of RA, as these semaphorins could reduce the inflammatory processes and joint destruction observed in the patients. In the case of Sema4A and Sema7A, the neutralization of these cytokines could be an interesting therapeutic approach for the treatment of RA and SSc. However, as Sema4A is involved in the expression of Th17 cytokines and Sema7A in the Th17 skewing, and both cytokines have a fibrotic role, they could be promising targets in Th17-mediated diseases (SpA, psoriasis), and in fibrotic diseases (pulmonary fibrosis, liver fibrosis). Finally, neutralization of Sema4D and Sema5A could be used for reducing inflammatory processes involved in RA pathology.

In conclusion, the role of semaphorins in rheumatic diseases is still largely unexplored, but different studies have shown that some of these family members are involved in disease pathogenesis, mainly RA and SSc. The existence of phase I clinical trials in solid tumors [83,84] and multiple sclerosis [85] (Clintrials.gov identifier NCT01764737; study completed), targeting Sema4D supports the importance of semaphorins in multiple diseases, but animal models and preclinical testing are needed to elucidate the potential use of semaphorins as therapeutic targets in rheumatic diseases.
